# A needle in the breast: a case report

**DOI:** 10.4076/1752-1947-3-7419

**Published:** 2009-07-22

**Authors:** Sami Karapolat

**Affiliations:** 1 Department of Thoracic Surgery, State Hospital, Bitlis, Turkey

## Abstract

**Introduction:**

A needle in the breast tissue is very rarely encountered in clinical practice.

**Case presentation:**

A 21-year-old woman presented with a needle in her right breast. She was operated on under fluoroscopic guidance. The needle was removed successfully.

**Conclusion:**

Because of the risk of migration of the needle into the thoracic cavity and adjacent vital organs, patients with a needle in their breast tissue must be treated surgically.

## Introduction

Needles embedded in soft tissue anywhere in the body are a common entity. However, a needle in the breast is an unusual manifestation, and because of non-specific clinical findings, it can be difficult to diagnose this condition clinically [1]. Thus, they may remain asymptomatic for many years.

## Case presentation

**Figure 1:**
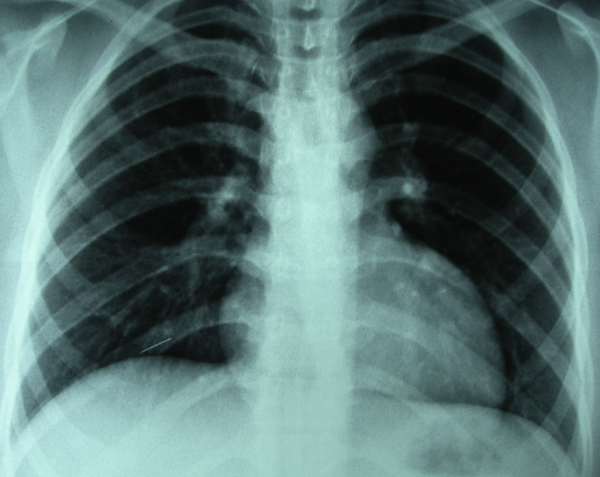
**Chest roentgenogram showing the needle in the right cardiophrenic sinus**.

**Figure 2:**
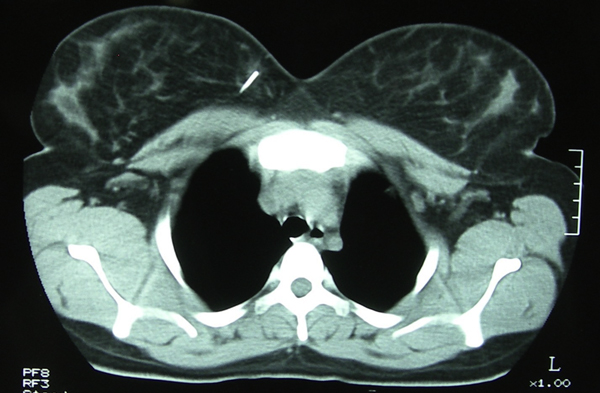
**Computed tomography of the thorax revealing the needle approximately 1**.5 cm in length in the right breast.

A 21-year-old woman was admitted with complaints of severe pain in her right breast and overlying skin bruising. Four years earlier, while she was receiving medical treatment for major depression, she had inserted a sewing needle into her left anterior chest wall to commit suicide and had told no-one. Blunt trauma had occurred on the right anterior chest wall approximately a week prior to presentation. During physical examination, sensitivity to palpation and an ecchymosis with a size of 4 × 3 cm localized in the upper left quadrant of her right breast were detected. A postero-anterior chest roentgenogram demonstrated a needle in the right cardiophrenic sinus (Figure 1). However, as the relationship of the needle to the intrathoracic cavity could not be clarified definitely with a lateral chest roentgenogram, a computed tomography scan of the thorax was performed. This showed a needle-like radiopaque object in the right breast tissue with no contact with the intrathoracic cavity (Figure 2).

**Figure 3:**
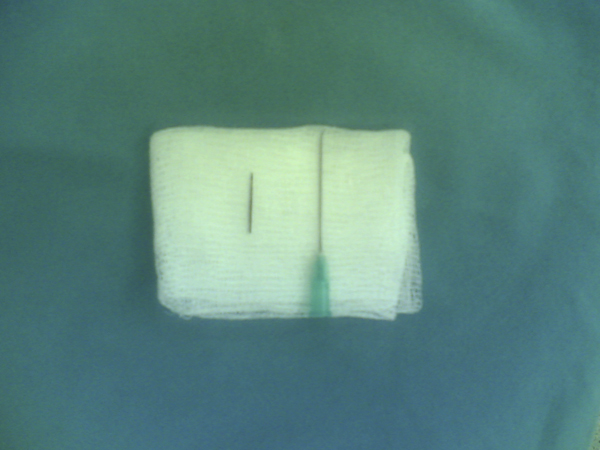
**Macroscopic appearance of the needle after removal**.

We made a 3 cm transverse incision on the right 4th intercostal space under local anesthesia, and the subcuticular fatty tissue was separated from the right breast tissue. At this stage of the operation, the location of the needle was detected definitely using C-arm fluoroscopy. It corresponded to the upper-middle part of the right breast tissue. Blunt dissection was performed and the needle, surrounded by intensive granulation tissue, was removed. It was observed that the needle was about 1.5 cm in length and rusty (Figure 3). Before closing the wound, an intra-operative chest roentgenogram was taken and no residual needle fragment was observed. Following hemorrhage control, the layers were covered appropriately. Recovery was uneventful and she was discharged from hospital two days after the operation. The patient has remained asymptomatic during six months of follow-up.

## Discussion

Cases of migrating needles in any part of the body may remain asymptomatic for a long time, or may be discovered through symptoms related to toxic and allergic reactions, inflammation, or to the development of an infection or an abscess. Having a high index of clinical suspicion, particularly in the absence of symptoms, is necessary for the diagnosis.

If the needle is small and localized deep in the tissue, it may be impalpable on physical examination if the patient is relatively obese, as was the case in our patient. It is necessary to be gentle and to avoid hard palpation during examination, otherwise the needle may migrate and penetrate into the pleural space or intrathoracic cavity and may lead to life-threatening complications, mainly pneumothorax.

Needles localized in subcuticular fatty tissue in any part of the body or in deeper tissues sometimes remain asymptomatic. Among these patients, it can be difficult to diagnose a needle localized in breast tissue, and the needle may be detected incidentally through routine chest roentgenogram examination. Our patient presented approximately four years after a suicide attempt and had remained asymptomatic during this period. However, as in our patient, blunt traumas occurring years later to the region where the needle lies may cause bleeding in the surrounding tissue, and this situation may reveal itself as a localized skin ecchymosis. Physicians encountering such situations should be clinically suspicious in order to reach a diagnosis, and then should obtain a careful and comprehensive anamnesis and investigate the region using radiography. Early diagnosis will prevent migration of the needle which may cause serious complications and lead to the need for complex therapeutic procedures.

Although it is known that needles embedded in the subcuticular layer of the chest wall can migrate towards the intrathoracic cavity, the lung parenchyma, the pericardium or the myocardium and thus may lead to life-threatening consequences such as pneumothorax, hemothorax, intrapulmonary abscess, cardiac tamponade, endocarditis and even death, the migration of a needle localized in the left anterior chest wall towards the right breast tissue is extremely rare [2]. We think that the needle migrated from the anterior of the sternum over time in our patient to the right breast tissue and remained in the soft tissue of her left anterior chest wall during the suicide attempt. We were led to this conclusion because the statement from the patient indicated that she decided not to commit suicide due to the severe pain as a result of the sewing needle inserted into her left anterior chest wall, and there was no evidence of another needle in the radiological examination we performed.

Removal of the needle from the breast is not an easy process. In particular, incisions which are made without determining the accurate site of the needle may result in unsuccessful interventions [3]. In general, it is difficult to locate needles in breast tissue. In order to achieve this, intra-operative ultrasound imaging may be the preferred method both because of ease of use and the fact that it allows staff members to avoid irradiation. Unfortunately, we were not able to employ this method in our clinic. Because of this, we used intra-operative C-arm fluoroscopy, and detected the location of the needle accurately and removed it successfully. Fluoroscopy helps in the accurate location of such radiopaque foreign bodies and facilitates their removal [4]. However, we think that real-time fluoroscopic examination may be helpful partly in order to search for residual fragments in the tissue after removal of the needle, but, because of its relatively low resolution, it cannot detect very small fragments. For this reason, as in our patient, a chest roentgenogram before the end of surgery is more useful in the detection of residual fragments. In a study by Sakai et al., it was reported that, after the removal of multiple needles located in the pericardium and lung, it is always necessary to obtain an intra-operative chest roentgenogram to avoid leaving residual fragments before closing the wound [5].

## Conclusions

A needle in the breast tissue should always be surgically removed as soon as possible even if the patient is asymptomatic, because of the strong possibility of developing an abscess and the risk of migration of the needle into the thoracic cavity, the lung or the heart. Intra-operative fluoroscopy will facilitate detection of a needle embedded in the breast tissue, and prevent unnecessarily long surgical intervention. It will thus reduce the failure and morbidity rates.

## Consent

Written informed consent was obtained from the patient for publication of this case report and any accompanying images. A copy of the written consent is available for review by the Editor-in-Chief of this journal.

## Competing interests

The author declares that he has no competing interests.

## Authors' contributions

SK carried out the operation, and prepared the manuscript.
